# Modeling information diffusion in social media: data-driven observations

**DOI:** 10.3389/fdata.2023.1135191

**Published:** 2023-05-17

**Authors:** Adriana Iamnitchi, Lawrence O. Hall, Sameera Horawalavithana, Frederick Mubang, Kin Wai Ng, John Skvoretz

**Affiliations:** ^1^Department of Advanced Computing Sciences, Institute of Data Science, Maastricht University, Maastricht, Netherlands; ^2^Department of Computer Science and Engineering, University of South Florida, Tampa, FL, United States; ^3^Department of Sociology, University of South Florida, Tampa, FL, United States

**Keywords:** social media, forecasting, data-driven, Twitter, Reddit, YouTube

## Abstract

Accurately modeling information diffusion within and across social media platforms has many practical applications, such as estimating the size of the audience exposed to a particular narrative or testing intervention techniques for addressing misinformation. However, it turns out that real data reveal phenomena that pose significant challenges to modeling: events in the physical world affect in varying ways conversations on different social media platforms; coordinated influence campaigns may swing discussions in unexpected directions; a platform's algorithms direct who sees which message, which affects in opaque ways how information spreads. This article describes our research efforts in the SocialSim program of the Defense Advanced Research Projects Agency. As formulated by DARPA, the intent of the SocialSim research program was “to develop innovative technologies for high-fidelity computational simulation of online social behavior ... [focused] specifically on information spread and evolution.” In this article we document lessons we learned over the 4+ years of the recently concluded project. Our hope is that an accounting of our experience may prove useful to other researchers should they attempt a related project.

## 1. Introduction

The last decade has seen the development and wide-spread adoption of social media platforms leading to a multitude of social, political, and economic implications, such as protest organization (Jost et al., 2018), spreading of socio-cultural movements (Mundt et al., 2018), adoption of risky behavior (Jaffar et al., 2020; Papadamou et al., 2021), disinformation (Gottlieb and Dyer, 2020), and content monetization (Goanta et al., 2022), to name only a few. Understanding how information spreads on such platforms is paramount not only for platforms, who are increasingly required to maintain an information environment free of malicious content (be it representations of violence, false information, scams, etc.), but for the society at large that saw its younger population threatened by bullying, screen addiction, and risky “challenges.”

Scientists have analyzed the spread of information in various contexts, such as political manipulation and disinformation (Starbird et al., 2019; Ferrara et al., 2020; Francois et al., 2021). Such studies focused on datasets of user interactions with online information and thus focused on a particular context defined by specific information topics, social media platform(s), and time period. But how would the same information spread if it was injected in the system at a different time, or on a different platform, or by a different user? Answering such questions can lead to stronger understanding of information spread on social media platforms, and could also open the way to designing efficient intervention techniques to limit potential damage. Modeling information spread is one way to reach such objectives. However, the challenges to develop an accurate, data-driven such model come from two main sources.

One such source is the differences in social media platforms that raise various challenges. First, social media platforms facilitate interaction with different types of content, from text-based micro-blogging messages (as in Twitter^1^) to pictures (Instagram^2^) to short or long videos (TikTok^3^, YouTube^4^). User engagement with content is different for a platform such as GitHub^5^, where users share code and raise issues related to software bugs, compared to a platform such as YouTube, where watching videos is the main activity. Second, platform features control user engagement with information. For example, YouTube acknowledged this fact recently and disabled the viewing of the number of dislikes^6^; Facebook never allowed a dislike (thumb down) button^7^; Twitter facilitates the spreading of messages via retweeting, while Reddit facilitates user engagement in a conversation via replies to posts. Third, platforms attract different types of audiences with different affinities for and habits of engaging with information and spreading it. For example, TikTok is more popular among younger audiences, especially teens, while Facebook is used by a wider range of age groups.^8^ This leads to different interests in online topics, and thus different topics to engage with and thus spread. Moreover, platforms are represented geographically in different ways: for example, Twitter along with other platforms is blocked in China^9^; local platforms such as Jamii are popular only in Africa.^10^ Fourth, platforms deploy their opaque content promotion algorithms that selects what information is presented to which user (Reviglio and Agosti, 2020). The contribution of these algorithms to information spread is difficult to isolate accurately in a changing technological and legislative environment. And finally, information spread is affected by customized attempts to exploit platform affordabilities and manipulate content promotion algorithms, as seen in various characterization of coordinated information operations (Varol et al., 2017; Choudhury et al., 2020; Ng et al., 2021b; Pacheco et al., 2021).

A second source of challenges in accurately modeling information diffusion online is the tight coupling of online and off line activities. Events in real life are visible online both in information spread and in attempt of manipulation. The recent COVID-19 pandemic led to increased activity on social media, and with it viral spread of (mis/dis)information related to vaccines, cures, and causes of the disease (Shahsavari et al., 2020; Islam et al., 2021; Muric et al., 2021).

This article describes our research efforts over about 5 years in developing data-driven social media simulations that accurately reflect the real user activities on different platforms and different socio-politico-geographical and informational contexts. Our project was part of the SocialSim program of the Defense Advanced Research Projects Agency. As formulated^11^ by DARPA, the intent of the SocialSim program, formally, Computational Simulation of Online Social Behavior, was “to develop innovative technologies for high-fidelity computational simulation of online social behavior ... [focused] specifically on information spread and evolution.” Over the course of the project, we designed and refined a series of machine learning-based models that accurately forecast engagement (as measured by number of users, posts, comments, etc.) with specific topics of discussion on different social media platforms. Our machine learning models (Ng et al., 2021a; Mubang and Hall, 2022) were trained per platform and per topic, thus implicitly learning intricacies of content promotion algorithms specific to each platform while simultaneously considering exogenous influences (e.g., real-word events) for predictions. In this article we document our lessons from developing machine learning-based simulators that accurately reflect information spread measured by various metrics on five platforms, six datasets, and accumulating more than 5 years of data. Our hope is that an accounting of our experience may prove useful to other researchers should they attempt a similar project.

The main conclusions of our effort can be summarized as follows: First, aggregate activity, such as the number of retweets on a particular topic within a particular hour, can be predicted with high accuracy over a future horizon of one or 2 weeks. However, we discovered that it is challenging—if at all possible—to make highly accurate predictions at finer granularity, such as user *U* will take action *X* at time *t*, using the limited information available through the social media platform APIs. In other words, a user's previous actions on the platform (that are typically sparse and occur in bursts) do not provide sufficient information to accurately predict future actions and their timing. On the other hand, the aggregated actions of multiple users in a particular topic can provide enough signal and historical information for machine learning algorithms to learn and make accurate predictions. Second, exogenous information, such as events in the physical world as captured in news, is required for accurate predictions as it often influences social media activity. For example, in geopolitical datasets, such as the Venezuelan political crisis of 2019, on-the-ground events (e.g., call for protests or armed confrontations) often drive a surge in engagement and online discussions in social media platforms. Third, performance metrics need to be matched to the prediction goals to get the most useful indication of utility. For example, average percentage error captures only part of the story. Depending on the practical use of the simulations, solutions with close to perfect average percentage error may show totally unrealistic patterns of social media activity. And finally, end-to-end machine-learning solutions yield limited performance due to the inherent compromises of satisfying multiple objectives; instead, decomposing the problem into specialized subproblems allows for easier reaching individual objectives and also for correcting anomalies.

The remainder of this paper is organized as follows. Section 2 describes the problem we address and overviews the existing literature that covers different aspects of the problem. Section 3 presents the scenarios and related data that we use to support our observations. The rest of the paper summarizes our lessons on designing social media simulators (Section 4), on how well-accepted performance measurements can be misleading (Section 5), on how much data is useful for training (Section 6), and on what features of social media can be realistically predicted (Section 7). We summarize the paper in Section 8.

## 2. Background

Our task was to develop a simulator that accurately models information diffusion within and across various online messaging platforms. Such a simulator should capture realistic online user activities at fine granularity—who responds to whom on which topic, and when—over a long time interval spanning weeks or even months. One way to test the veracity of such a simulator is as a forecaster: use a training dataset to parameterize the simulator, obtain the output of the simulator for the (testing) period following the training period, and compare that output with the ground truth data.

### 2.1. Problem definition and setup

Formally stated, our task was to forecast a series of events of the form < *user, message, action, time*> where *user* is the (anonymized) identifier of a user in the dataset previously seen for training, *message* corresponds to either a topic or a token such as a specific URL, *action* represents the specific messaging actions on a particular platform, such as tweet/retweet/quote/reply on Twitter or post/comment on Reddit, and *time* is the timestamp of the predicted event. When *action* is in response to a previous message (for example, a retweet), that previous message should also be specified by its message identifier.

We had access to anonymized datasets for training with a similar format as the one desired for output. We used multiple datasets collected using public APIs from platforms such as Twitter, YouTube, and Reddit, augmented with information resulting from topic and sentiment analysis. In addition, we used various other publicly available datasets such as GDELT and ACLED as records of events in the physical world that might catalyze online discussions. As typical to DARPA-funded projects, our solutions were tested twice a year during the famous DARPA challenges on real, previously unseen data. It is this rigorous evaluation coupled with access to rich collections of data from multiple social media platforms that led to the lessons described in this paper.

### 2.2. Related work

Recent studies have proposed end-to-end solutions attempting to capture various macro and microscopic characteristics of information diffusion processes (Section 2.2.1). Other researchers focused on specialized, smaller tasks: timeseries forecasting for predicting the number of messages on or users engaged with particular topics over time (Section 2.2.2), prediction of information cascades (Section 2.2.3), and prediction of user interactions (Section 2.2.4). We discuss each of these topics below.

#### 2.2.1. End-to-end simulators

Most of the recent attempts in modeling fine grained social media activity come from the DARPA SocialSim program. Simulation approaches in this context have particularly focused on two design choices: bottom-up and top-down.

Bottom-up solutions simulate social behavior in online environments by modeling individual users' actions and their interactions within a diverse population. Agent-based-modeling (ABM) approaches are popular in this space, where hand-crafted rule sets are built to model agents' behavior and their interactions. Bhattacharya et al. (2019) proposed an ABM that incorporates social and cognitive agents that model online human-decision making. Garibay et al. (2020) proposed DeepAgent that can simulate social dynamics in a multi-resolution setting at the user, community, population, and content levels. Murić et al. (2022) developed an ABM that provides support for simulations of cognitive behavior and shared state across multiple compute nodes. They augmented the agents' decision-making process by employing machine learning models that predict the probability of interaction between an agent and a particular resource. They showed their models can outperform simple probabilistic models, in which agents decisions are based on frequencies in the training data. Yao et al. (2018) proposed SocialCube, an agent-based approach with self-configurations based on past social media traces, which allows for recomputing the probabilities of individual action types. While ABM approaches can provide valuable insights of information spread such as detailed interplay between individual behaviors and network structure, they do not scale well with large populations. Modeling the behavior of new users is yet another challenge for most ABM techniques as agents often rely on historical records/interactions to make decisions.

In contrast, top-down simulation approaches focus on modeling the dynamics of a population or community as a whole based purely on data-driven decisions. The idea is to divide the problem into sub-components, where microscopic (i.e., user interactions) and meso-level properties (i.e., cascades predictions) can be guided by macroscopic predictions that capture volume, audience and timing of events (Liu et al., 2019; Horawalavithana et al., 2022; Mubang and Hall, 2022). Despite limiting the microscopic predictability of the simulations due to accumulation of errors, these methods can easily scale to simulate massive populations and do not rely on complex sets of rules. In this article, we describe our experience with developing top-down approaches for social media simulations of information diffusion.

#### 2.2.2. Timeseries forecasting

Traditional approaches used for forecasting-related tasks in social media contexts include the Autoregressive Integrated Moving Average model (ARIMA) (McClellan et al., 2017; Abdelzaher et al., 2020), Exponential Smoothing (ES) (Odlum and Yoon, 2015; Hong et al., 2017), and Hawkes Processes (Pinto et al., 2015; Zhao et al., 2015; Rizoiu et al., 2017). These approaches typically suffer from limitations when presented with complex and irregular temporal trends, which is usually the case with social media timeseries (Zhang et al., 1998; Bacry et al., 2020). Despite their limitations, these models are commonly used as baselines in social media temporal-based problems (Shrestha et al., 2019; Tommasel et al., 2021; Mubang and Hall, 2022; Ng et al., 2022).

Machine learning techniques are often preferred over traditional approaches, in social media forecasting, due to their ability to model non-linear behavior. Several deep learning methods such as Convolutional Neural Networks (CNN) and Recurrent Neural Networks (RNN), as well as machine learning algorithms like gradient-boosted decision trees, have been proposed for timeseries forecasting in literature. Hernandez et al. (2020) applied graph convolutional networks for forecasting user activity timeseries. Abdelzaher et al. (2020) proposed a deep learning technique that combines both CNN and RNN to forecast individual user activity streams. Both studies showed the challenges of forecasting at the level of individual user accounts due to the heterogeneity and often sparsity of their activities. However, both highlighted that forecasting the activity streams of groups of users results in a more feasible and tractable task. Many studies in the social media domain attempt to forecast the activity of content entities such as topics, hashtags (Kong et al., 2014), or keywords (Saleiro and Soares, 2016) shared on online messages. Liu et al. (2019) explored several machine learning methods to predict whether and when a topic will become prevalent. The authors highlight the challenges faced in forecasting the frequency of topics discussed by users due to irregular patterns. Shrestha et al. (2019) used a deep learning recurrent model to forecast the number of retweets and mentions of specific news sources on Twitter using the network structure observed in the day before the predictions. They found that small but dense network structures are helpful in predictions.

Most studies in social media timeseries forecasting have been applied to short-term settings (i.e., predicting only the next day of activity). Yet a more challenging task is that of forecasting for longer periods of time, where immediate historical activity from social media platforms is not available during inference. For this task, considering both endogenous (i.e., internal activities in the platform) and exogenous (e.g., real-word events, or activities from other online sites) signals is paramount for accurate predictions. For example, Dutta et al. (2020) predicted the volume of Reddit discussions in a future horizon leveraging the text from news and an initial set of comments using a recurrent neural network architecture. The study corroborated the superiority of models that consider exogenous signals through a series of ablation studies. Horawalavithana et al. (2019) and Liu et al. (2020) showed that the volume of activities on Twitter and Reddit carry important predictive power to forecast the activity on GitHub related to common vulnerabilities and exposures security issues.

#### 2.2.3. Cascade prediction

The goal of cascade prediction is to model conversation trees and their evolution over time. Most of the previous work has been focused on modeling and predicting properties of individual cascades (Gao et al., 2019). To predict the size of a cascade, several solutions have been proposed using statistical approaches (Liben-Nowell and Kleinberg, 2008), while others used machine-learning methods with domain-specific features (Cheng et al., 2014; Yu et al., 2015). Previous work has shown that predicting the popularity of a cascade is not trivial (Gao et al., 2019). However, when given some initial conditions about the initial state of the cascade, multiple dimensions of message popularity can be accurately predicted (e.g., the final cascade size, shape, virality, etc.). Based on this understanding, several deep learning methods have been proposed for this problem. Embedded-IC (Bourigault et al., 2016) embeds cascade nodes in a latent diffusion space to predict the temporal activation of a node. DeepCas (Li et al., 2017) proposed a diffusion-embedding framework to predict the incremental growth of a cascade. DeepDiffuse (Islam et al., 2018) and DeepInf (Qiu et al., 2018) utilized the underlying cascade structure to predict the future node activation in a cascade using the recurrent neural network. Specifically, DeepDiffuse predicts the user and the timing of the next infection, but does not predict the evolving cascade structure. They also assume any node can be infected once during a cascade.

Most of these previously mentioned studies focused on classifying viral cascades, or predicting the future activation of a node over discrete time intervals, but do not focus on modeling the evolving cascade structure. More relevant to our task is the work of Zayats and Ostendorf (2018), who proposed a graph-structured LSTM model to capture the temporal structure of a conversation. They show the effectiveness of the model on predicting the popularity of Reddit comments. Other proposed techniques that predict the popularity of conversations are mostly based on statistical approaches that have focused on predicting an individual tree structure (Wang et al., 2012; Aragón et al., 2017; Medvedev et al., 2017; Ling et al., 2020). These data-driven models attempt to capture and interpret some interesting phenomena of a given dataset, by estimating statistical significance of different features related to human behavior, in contrast to the fully data-driven models. The predictive performance of these parsimonious models may deteriorate due to the dependence on the chosen parameters and optimization of the likelihood function. Further, these models lack the capability of mapping users and exact timing information to the internal nodes.

While significant work has focused on predicting individual cascades, less attention has been invested in predicting the popularity of a group of cascades. For example, several works predict the aggregate volume of user activities on Twitter via Hawkes processes that model the events around a group of cascades (Valera and Gomez-Rodriguez, 2015; Zarezade et al., 2017). Theoretical models that capture the spread of social-influence when a group of competitive cascades evolve over a network have also been proposed (He et al., 2012; Lu et al., 2015). Other works have made similar observations when exploring inter-related cascades in multiplex networks (Xiao et al., 2019). These studies stress the importance of focusing on a group of cascades instead of an individual cascade for improving the prediction results of user-level diffusion behavior. Horawalavithana et al. (2022) proposed a data-driven machine learning model to predict conversation trees with author identities and continuous timing information mapped onto the nodes in the tree. Their solution captures the relationship between different microscopic conversation properties such as structure, propagation speed, and users who participate in a set of simultaneous cascades.

#### 2.2.4. User interaction prediction

User interaction prediction refers to the task of mapping user identities or categories of users (e.g., old users vs. new users) to predicted links of a given interaction network or cascades. In other words, the goal is to predict which user or type of user responds to whom. There have been several previous works proposed for this task. Some utilize embedding techniques, in which neural networks, typically graph neural networks (GNN), are used to embed each node in a given network into a low dimensional space (Perozzi et al., 2014; Grover and Leskovec, 2016; Goyal et al., 2020). These embeddings can then be used for node classification or prediction tasks as they encode useful information about a given node and its neighborhood. Despite the recent success of GNN for link prediction tasks, they have limitations. Xu et al. (2018) showed that GNN methods are not significantly more powerful than simple neighborhood aggregation graph kernels such as Weisfeiler-Lehman. Huang et al. (2020) found that combining label propagation and feature augmentation techniques can outperform GNN performance on a variety of datasets. Another concern is due to the quality and lack of diversity of the benchmark graph datasets that are typically used to evaluate these methods.

Other non-neural network approaches such as matrix factorization (Dunlavy et al., 2011; Ma et al., 2017) or probabilistic methods (Sarkar et al., 2014; Ahmed and Chen, 2016) have also been proposed for this task. However, these methods often suffer from computational complexity in terms of space and time. Perhaps, the major limitation of many of these traditional predictive models is that they are built only for nodes who are already seen in the training data (transductive capability), but not for new nodes (inductive capability), which are important in social networks where activities are strongly driven by new users. While recent deep learning methods attempt to overcome this issue (Hamilton et al., 2017), it still remains a very challenging problem, especially in online social networks. With these challenges in mind, we attempt to combine both discriminative (machine learning) and generative approaches to model user interactions that (1) consider the appearance of new users and (2) attempt to capture important structural properties of message cascades.

## 3. Datasets

As a data-driven project, we had access to a rich collection of curated online data on a variety of contexts ranging from cyber-security vulnerabilities and crypto-currency schemes to disinformation campaigns and geo-political events. We briefly describe below the datasets used in this article to support our observations.

**Venezuelan Political Crisis of 2019 (Vz19):** In 2019, a humanitarian, economic and political crisis engulfed Venezuela as the presidency was disputed between Juan Guaidó and Nicolás Maduro, each claiming to be the country's rightful president.^12^ The political turmoil had its roots in the controversial re-election of Nicolás Maduro as the head of the state on January 10th, which was boycotted by opposition politicians and condemned by the international community as fraudulent. This event contributed to unprecedented conflicts and high political tension which resulted in nationwide protests, militarized responses, international humanitarian aid intervention attempts, and incidents of mass violence and arrests. The dataset consists of records from two online platforms, Twitter and YouTube. The Twitter dataset includes 16,624,066 activities done by 1,140,865 users. The YouTube dataset includes 146,337 activities done by 85,049 users. The evaluation scenario consisted of training data from December 24, 2018 to February 21, 2019 (1 month and 28 days) and testing data from February 22 to April 1, 2019 (1 month and 7 days).

**Chinese-Pakistan Economic Corridor (CPEC):** CPEC is a strategic economic project under the Belt and Road Initiative (BRI) launched by China aimed at strengthening and modernizing road, port and energy transportation systems in Pakistan. While China's investment in Pakistan has largely taken the form of infrastructure development, the project has been heavily criticized with claims of lack of transparency, self-benefit and imposing unsustainable debt on the Southeast Asian country.^13^ Conflict around this project plays out in discussions on social media, where state actors strategically promote Chinese interests to facilitate building projects and garner further investment. The dataset consists of records from three online platforms, Twitter, YouTube, and Reddit. The Twitter data consists of 5,248,311 activities done by 1,216,100 users. The YouTube dataset consists of 296,625 activities done by 146,705 users. The Reddit data consists of 645,394 done by 184,836. The evaluation scenario consisted of training data from March 1 to May 28, 2020 (2 months and 27 days) and testing data from May 29 to August 1, 2020 (2 months and 3 days).

**Belt and Road Initiative in East Africa (BRIA):** As part of the same BRI flagship project, China has committed a substantial amount of resources and investment across Africa, especially in the east African nations. Similar to the CPEC scenario, several concerns have arisen over China's strategic intentions in Africa. The dataset consists of records from three different online platforms, Twitter, YouTube, and Jamii.^14^ The Twitter data consists of 1,832,156 activities done by 705,042 users. The YouTube data consists of 20,289 done by 14,429. The Jamii data consists of 18,058 activities done by 3,221 users. The evaluation scenario consisted of training data from February 1 to March 31, 2020 (2 months) and testing data from April 1 to May 1, 2020 (1 month).

**Data Augmentation:** One of the objectives of the SocialSim program was to investigate and simulate how social media activity diffuses for particular pieces of information, which throughout this paper we refer to as *topics*. Over the course of the project, these topics were represented in various ways, from simple terms or keywords to more complex and manually crafted narratives. For example, in the cybersecurity-related datasets, the topics were simply identifiers for security vulnerabilities. Similarly, the crypto-currency datasets were tracking keywords naming crypto-currencies, such as beancash, agrello, electroneum, indorse, etc. The White Helmets dataset used the notion of narratives, which were defined as a set of keywords that can coherently describe a particular event. These narratives were created using a combination of topic modeling algorithms, such as Latent Dirichlet Allocation (LDA) and Non-negative Matrix Factorization (NMF), and event extraction methods. More details on this narrative extraction framework can be found in Blackburn et al. (2020). For the *Vz19, CPEC*, and *BRIA* datasets, the topic assignment strategy was identical. Specifically, subject matter experts (SME) were employed to identify relevant sets of keywords relating to each context. SMEs created an initial annotated set of data comprised of a small subset of social media posts, which were labeled with their corresponding topics. For each challenge, a BERT language model was fine-tuned on the manually annotated corpus for the topic annotation task, and eventually applied to the set of non-labeled posts. More details about the annotation process and accuracy of the trained BERT models for these datasets can be found in Ng et al. (2023).

Due to the limitations of the Twitter API, information regarding direct retweets between users cannot be inferred at collection time. Thus, the complete cascade of retweet interactions (i.e., retweets of retweets) is not available. However, using Twitter's follower graph, it is possible to infer the correct retweet path of a tweet. The code to reconstruct the retweet cascades given a set of original tweets is publicly available in GitHub.^15^ This code is a re-implementation of a retweet reconstruction algorithm proposed in Vosoughi et al. (2017). The Twitter follower API was used to get the list of followers for all users who appeared in each of the Twitter datasets we worked with.

## 4. Lessons on the design of social media simulators

During the duration of project, the design of our online social media simulators (OSMS) drastically changed from attempting to capture all aspects of the problem with a single end-to-end solution to breaking down the problem into smaller, modular components that more accurately capture dimensions of the problem. These choices were driven by our successes and failures during the challenges and by data-driven observations, which are summarized by the following lessons learned.

### 4.1. From end-to-end solutions to a decompositional approach

In early stages we experimented with various frameworks for simulating social media behavior in an end-to-end manner, where the main objective was to capture micro-level user interactions at the finest time granularity. Specifically, we wanted to predict which user will act on what information at which hour. One of our solutions used sequence-to-sequence LSTM models to directly predict the number of activities a user would perform in future timesteps (Liu et al., 2020). Another approach consisted on leveraging more complex ML-architectures, specifically diffusion convolutional recurrent neural networks, which aimed to combine both social network information and historical time series to predict the future activities of users (Hernandez et al., 2020). While these approaches seem to work well compared to several baselines, there were still a number of limitations and challenges in predicting user activity, some of which are related to dataset characteristics specific to social media behavior.

**Figure 1:**
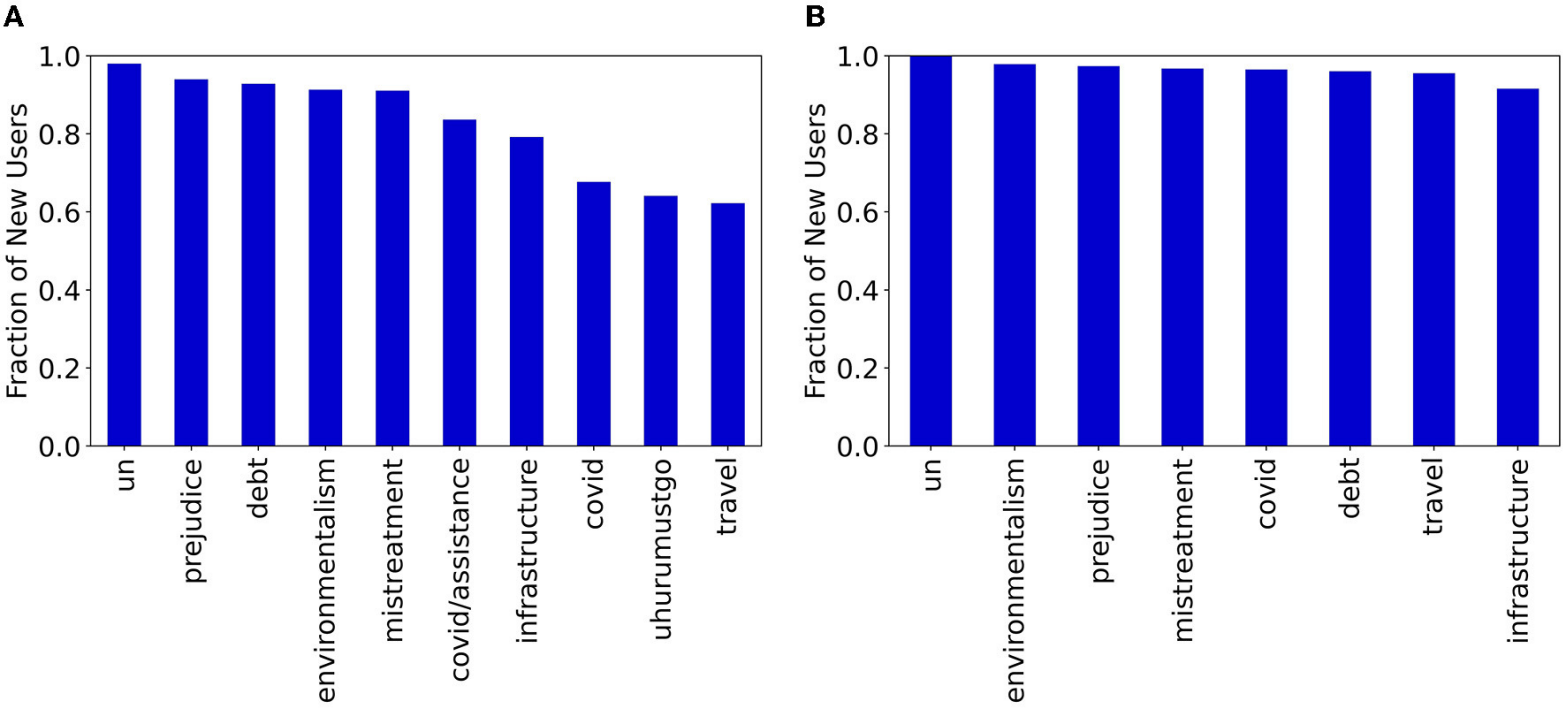
Fraction of new users during the testing period not previously seen in the training period for BRIA topics across Twitter and YouTube. **(A)** Twitter. **(B)** YouTube.

One challenge was the significant sparsity present in user activities. As shown in previous studies (Barabasi, 2005; Qiu et al., 2018), users on social platforms often operate in a heterogeneous and bursty fashion, where active periods are followed by very long periods of inactivity. Based on data-driven analyses, we observed that more than 75% of users in both Vz19 ad CPEC datasets have an average weekly activity rate of zero. This highlights that for most users, our models need to deal with really sparse time series data. The sparsity of user historical data poses a major problem for many forecasting approaches as it encourages models to severely under-predict the actual number of activities. Another limitation is that our end-to-end solutions do not account for new user engagements. Data analysis shows that in certain topics there is a considerable number of new users that make up a large portion of activities in a given day. Figure 1 shows the fraction of new users for different topics of discussion and across different social media platforms in the BRIA dataset during the testing period. In Twitter, at least 60% of users who interact are new, where “new” in this case means not previously seen in our training data. In YouTube, the fraction of new users who interact each day is much larger (at least 90%) for all topics. For this reason, it is necessary to account for new users when modeling social media activity, as they are the engaged user accounts.

**Figure 2:**
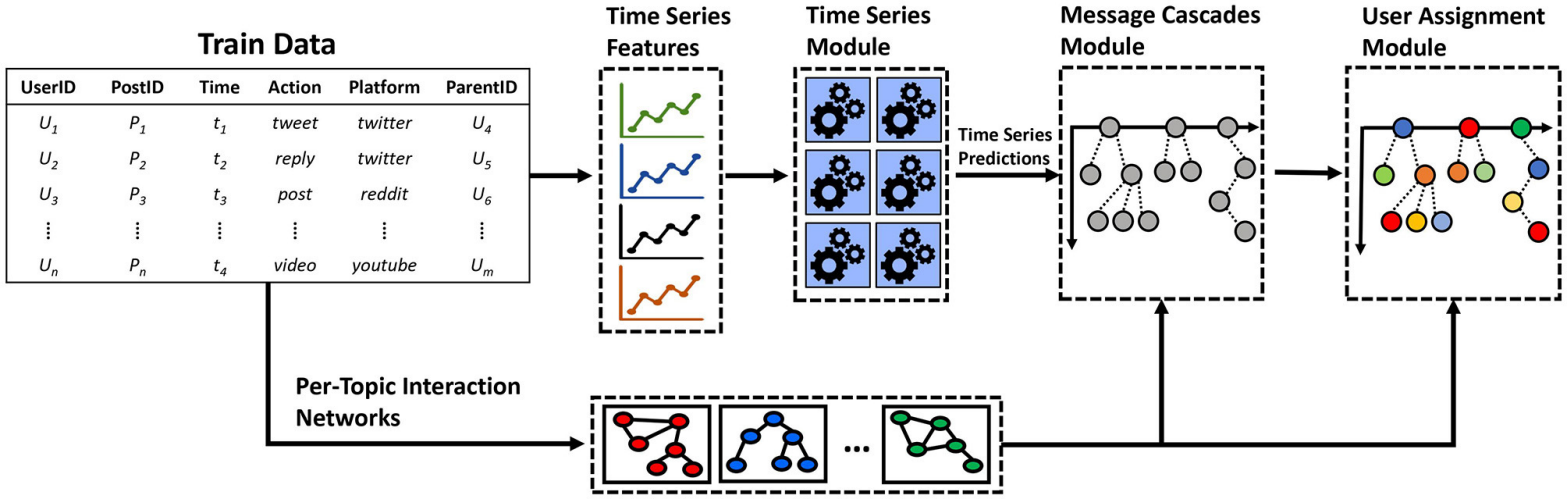
Framework for the modular design approach we proposed in the SocialSim challenges. Time series features are extracted from the train data and are used for regression tasks predictions such as activity counts, new users, old users, etc. The predicted time series along with social interaction networks are used to generate conversation trees in the message cascades module. Finally, user identities are assigned to the nodes of the conversation trees in the user-assignment module.

These data-driven observations encouraged us to depart from an end-to-end, monolithic model and adopt a decompositional approach, where we could use trained models for specific aspects of the problem. Figure 2 shows the final design of our model pipeline, which consists of three main components. First, we implemented specialized time series forecasting models that were tasked with different objectives such as predicting the number of activities (e.g., number of tweets, retweets, comments, etc.), or the number of new and the number of previously-seen users. We experimented with various ML algorithms, such as LSTMs and XGBoost regressors, which achieved good performance against traditional baselines such as ARIMA or Shifted, as shown in Mubang and Hall (2022) and Ng et al. (2022). We emphasize that the time series algorithms we used can be easily substituted for any other regression-based model of interest.

The second component in our pipeline was tasked with generating conversation trees. That is, given a set of predicted activities per unit of time (in our case, hour), the objective is to distribute such activities to individual information trees in order to model which message is a response to which (in a Reddit conversation, for example) and eventually forecast the evolving network structure of social interactions by assigning users to such messages. To this end, we leveraged a conversation pool generation algorithm, which uses branching processes to model the structure of social media conversations. More details on the specific algorithm can be found in Horawalavithana et al. (2022).

The final component in our pipeline consists of user assignments, where the task is to assign social media users to the conversation nodes of the predicted information cascades. One of our strategies framed this problem as a temporal link prediction task, where we used diffusion probability tables—inferred though temporal user-interaction networks—to predict which user was most likely to interact with whom. More details on this user-assignment methodology can be found in Mubang and Hall (2022). This divide-and-conquer simulation framework showed a significant improvement over our previous solutions in the various metrics that were considered during the SocialSim challenges.

### 4.2. Specialized time series forecasting models work better than one single model

During the several iterations of our model design, we learned that one single ML model trained for the task of time series forecasting is not sufficient to capture all complexities and changing relationships between topics that occur within the same social media platform and on different platforms. The design choices in our time series forecasting component were based on several data-driven observations, which are summarized by the following lessons.

**Figure 3:**
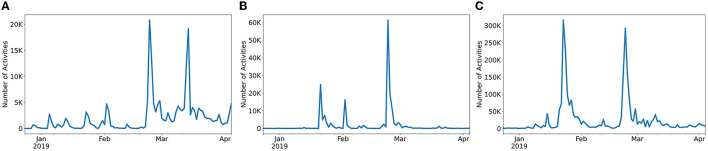
Time series of activities for three Twitter topics in the Vz19 dataset. The plot highlights the different scale of activity (topic popularity) on different topics on the same social media platform. **(A)** Maduro/cuba_support. **(B)** Military/dessertions. **(C)** Violence.

First, we learned that activity of different topics differs widely on the same platform. Topic popularity within the same social media platform rapidly changes based on user interest and current social trends. Topics that had previously seen a wide audience engagement might quickly die out as newer topics arise. Figure 3 shows the time series for three topics of discussion in Twitter during the Vz19 political crisis. We observed that peaks of activity for each topic often occur at different times either due to users interest or correlations with on-the-ground events. We also observed that different topics tend to show widely different magnitudes of activity. For example, the activity on the *violence* topic is 13X larger than in *maduro/cuba_support*. Due to these observations, we opted for a per-topic model design, where independent ML models are trained for each topic of discussion.

**Figure 4:**
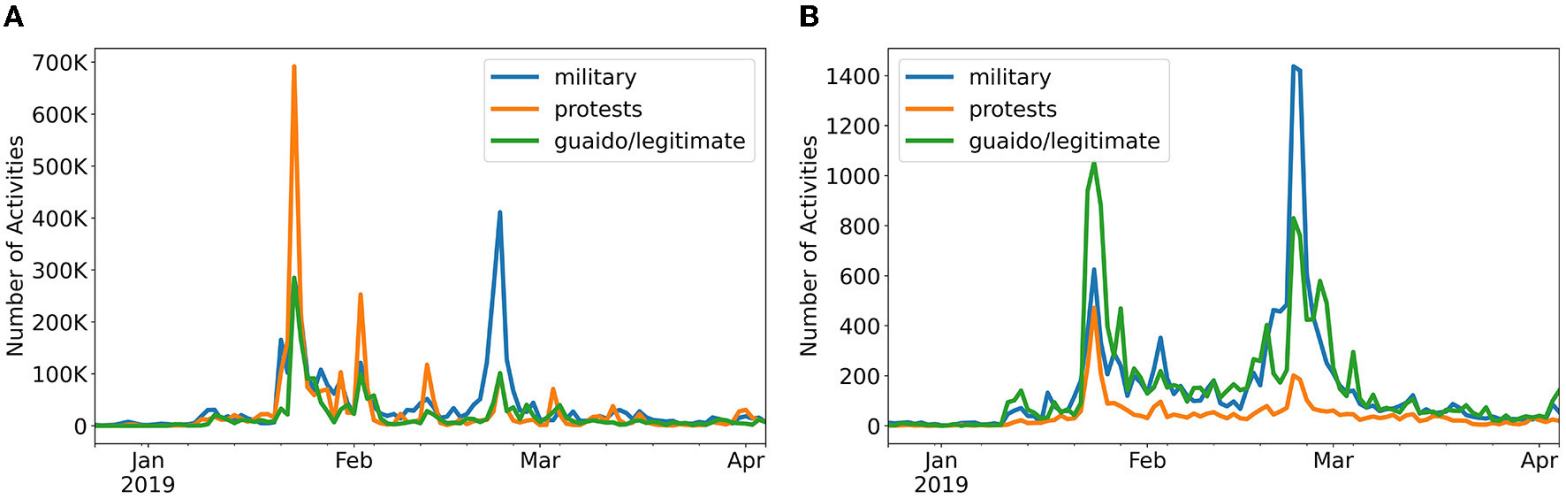
Time series of activities for three topics in the Vz19 dataset across two different platforms. The plot highlights the different scale of activity on the same topics but different social media platforms. **(A)** Twitter (Vz19). **(B)** YouTube (Vz19).

Second, we observed that the same topic of discussion can have different volumes and patterns of activity on different social media platforms. In Figure 4, we compared the time series for three Vz19 topics across two different platforms: Twitter and YouTube. We observed that there are clear differences in the magnitude of activities across the two platforms. Twitter activities are significantly larger than YouTube as shown by the order of the magnitude differences between topics being in the hundredths. These differences are expected due to the nature of each platform. Twitter is a micro-blogging service with features for large-scale distribution of information while YouTube is a video-hosting platform where large-scale interactions are not expected. Bearing in mind these differences, we decided to include specialized per-platform models as an attempt to capture such contrasting behaviors.

**Figure 5:**
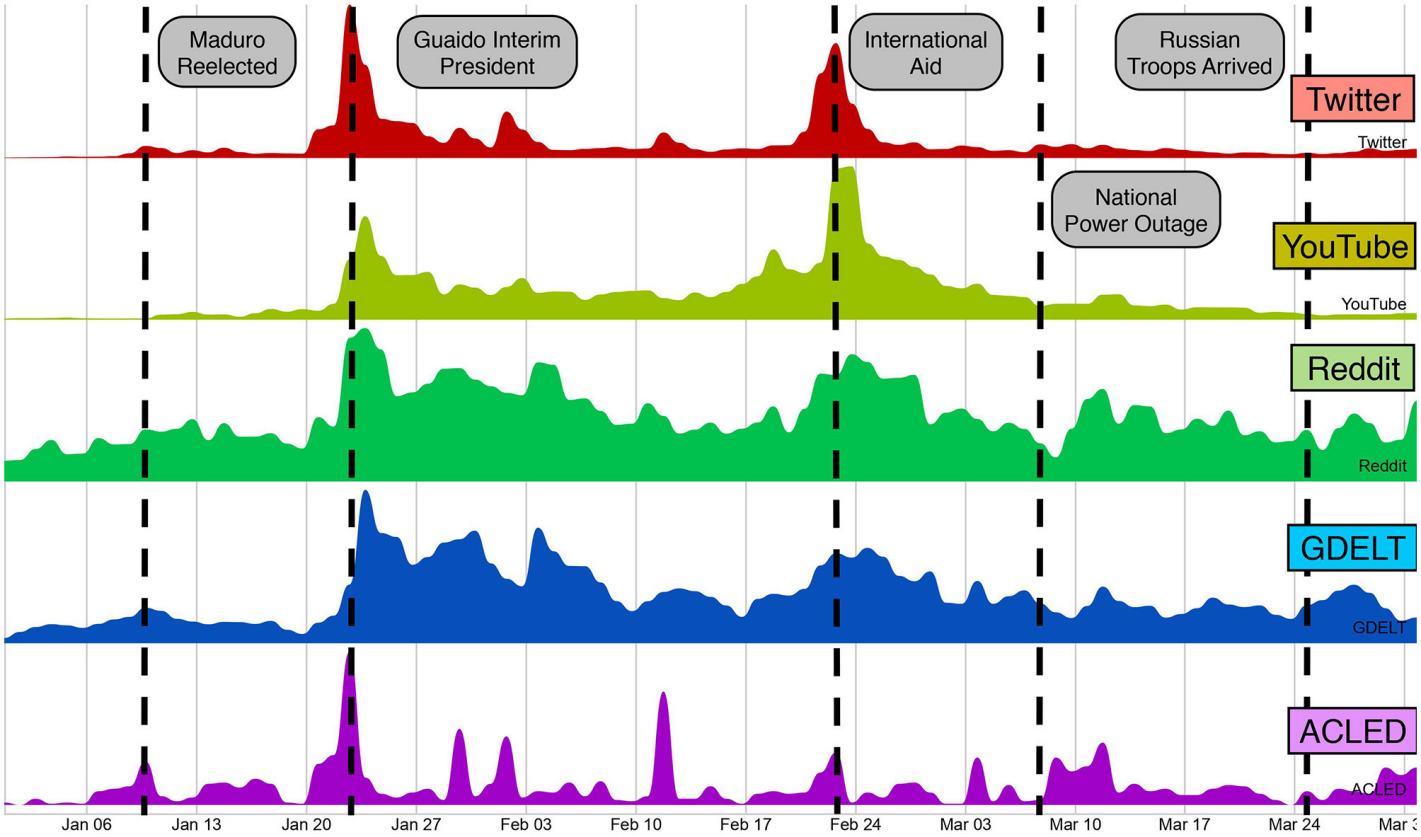
Time series of activities related to the political crisis events in Venezuela during 2019 across different social media platforms and exogenous sources. The figure shows some correlation between social media activities and external real-world events as recorded by two geo-political databases: GDELT and ACLED.

Third, online discussions are often correlated with different exogenous signals over time, especially in datasets driven by influential events, as shown in Dutta et al. (2020) and Horawalavithana et al. (2021). Figure 5 shows that, during the Venezuelan political crisis in 2019, social media activity from different platforms was often correlated with particular external events and news articles reports recorded in geopolitical databases. However, not all external events have a direct influence on a particular online topic. To capture the changing relationship between different online topics and exogenous sources, we proposed in Ng et al. (2022) a technique that dynamically selects models trained with different exogenous signals to better predict the future activity of a particular topic.

### 4.3. Correction of errors across multiple sub-components is necessary

The modular design as compared with the monolithic solution comes with a trade-off between performance and complexity. While the modular design made the problem more manageable, it also requires lots of supervision. As outputs from earlier components flowed down the pipeline, it was necessary to correct errors that could negatively impact the final simulation results. For example, during our work on different datasets, it became clear that accurate macro-level prediction modules (e.g., components that predict the volume of activity or the number of users) set the tone for more accurate micro-level simulations. Hence, we designed specialized modules to predict the number of posts and interactions over time for the duration of the forecasting window. As these predictions stay closer to the ground truth, our cascade generation module produces more accurate information cascades.

Table 1 illustrates the importance of accurate macro-level predictions and their impact on network-level measurements such as the Relative Hausdorff (RH) distance, which measures the similarity between user in-degree distributions of two networks. The results clearly show that accurately capturing the volume of activities over time has a significant impact on network measurements. For example, if the ground truth volume is input to the cascade model, our solution outperforms the shifted baseline in 8 out of 10 topics. The number of edges generated by our cascade process was controlled by the number of interactions predicted, so the smaller the error in these predictions, the smaller the error in the constructed networks.

**Table 1:** RH distance results for topics in BRIA dataset for two model variations and the shifted baseline.

**Topic**	**GT-model**	**NO-GT-model**	**Shifted baseline**
COVID	**0.58**	0.86	1.12
COVID/assistance	**0.77**	0.99	0.80
Debt	**0.72**	2.56	1.29
Environmentalism	**0.62**	0.88	1.00
Infrastructure	1.22	0.54	**0.31**
Mistreatment	**0.93**	2.61	1.50
Prejudice	2.64	2.81	**0.50**
Travel	**0.93**	1.13	1.00

GT-Model takes the ground truth volume of activities as input while NO-GT-Model takes the volume of activities predicted by a trained internal model (i.e., uses only historical platform activity as features). Both model variations generate cascades and user interactions based on a branching model.

Bold indicates best model.

Additionally, our pipeline carried out multiple post-processing checks to prevent generating events or predictions that were unrealistic to the social phenomena/behavior we modeled. For example, we make sure that the number of users who tweet was not larger than the number of tweets predicted by our time series forecasting components. We also checked that predictions with our ML components matched particular patterns observed in social media platforms (for example, the number of tweets being typically smaller than the number of retweets, or number of users being less than or equal to the number of interactions). This lesson highlights the importance of designing accurate macro-level components and regularly correcting errors that can negatively affect the final model performance.

### 4.4. Previous user reactions are more telling than previous user interactions

**Figure 6:**
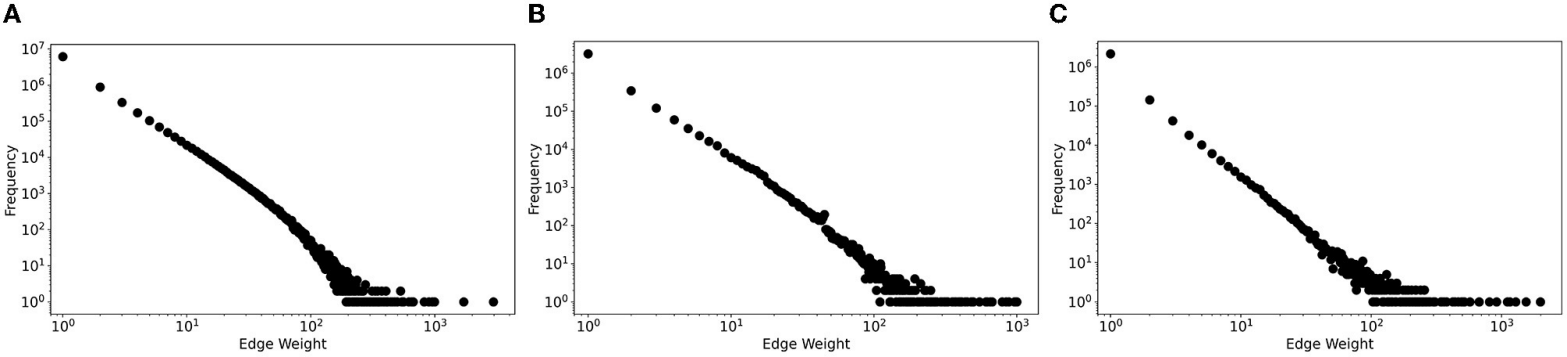
Log-log edge weight distributions of Twitter interaction networks across three different datasets. **(A)** Vz19. **(B)** CPEC. **(C)** BRIA.

In our early user-assignment strategy, we exploited the social network topology of previous user interactions (Horawalavithana et al., 2022) in an attempt to capture the well-accepted observation according to which people who interact more often will tend to interact more often in the future as well. Specifically, we extracted, for each topic, an interaction network where nodes are users and edges are weighted by the number of previous interactions to bias the assignment of users to predicted messages. One limitation of this strategy is that it did not account for the fact that in many cases the bulk of users interacting with a particular message were most likely new users, who had never been seen in the interaction networks. In fact, from data analysis, we observed that only very few pairs of users repeatedly interacted with each other as shown by the edge weight distributions in Figure 6. We found that at least 75% of pairwise user interactions have an edge weight of one during the training period, which was approximately two months for all datasets listed in the figure. Such observation holds across all social media datasets we worked with.

Thus, we changed our strategy and assigned users to cascades based on their susceptibility of being retweeted, thus changing the focus from edges to nodes in the social network of user interaction. We considered two user archetypes: (1) spreaders, those whose retweet to a tweet often attracts other retweets; and (2) frequent retweeters, those whose retweets are not retweeted by others. The spreaders were assigned to branch nodes, and the frequent retweeters were assigned to leaf nodes. The predicted number of new users from previous components are also assigned to the frequent retweeters user category as new users mostly engage in retweeting rather than posting. These modifications significantly improved our performance against several baselines in network metrics.

## 5. Lessons on performance measurements and baselines

Performance metrics are integral to evaluation in order to measure success. To evaluate the fidelity of simulations of social behavior a set of metrics was developed by researchers at Pacific Northwest National Laboratory (PNNL). These metrics were intended to cover three different levels of the simulation phenomena: node-level (includes predictive metrics for individual users such as predicting whether a user will propagate a message), community-level (includes measurements associated with a users or messages), and population-level (includes measurements for the full diffusion process such as quantifying the total number of messages shared; Saldanha et al., 2019). The implementation for this wide range of metrics is publicly available in PNNL's GitHub repository^16^. The lessons we describe in this section focus particularly on population-level performance measurements, especially those related to time series forecasting tasks. However, we emphasize that such lessons can also be applicable to evaluation efforts that focus on other levels of the diffusion phenomena.

**Figure 7:**
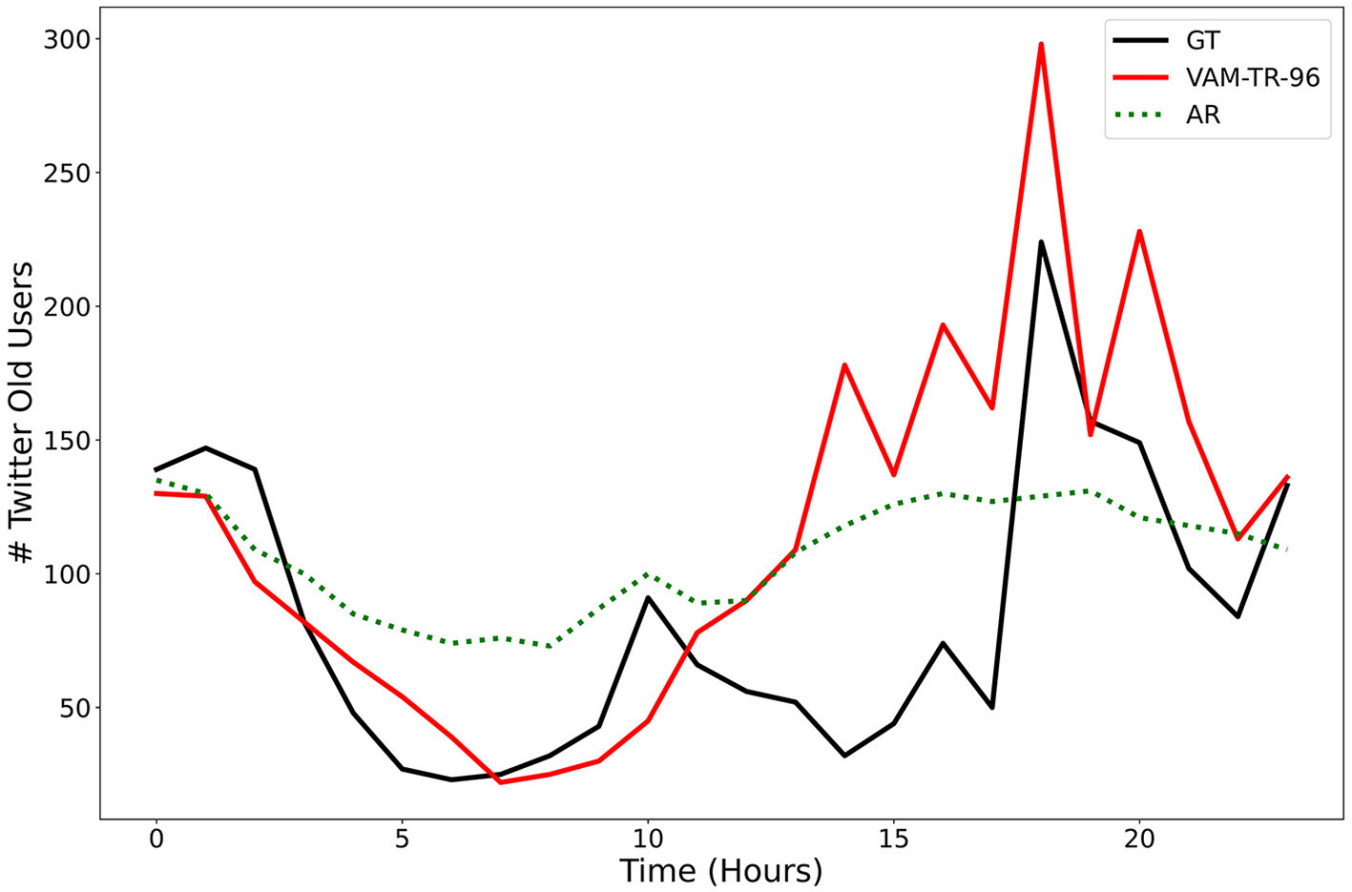
The topic *maduro/narco* from the Vz19 dataset on Twitter. The red curve is the XGBoost prediction, the dotted green curve is the AR prediction (baseline), and the black curve is the ground truth. Although the AR model had a slightly lower RMSE and MAE than XGBoost, XGBoost was able to capture a spike that occurred around 18–19 h. This shows that the selection of metrics should depend on the intended phenomenon to capture.

First, the identification of the right set of time series forecasting metrics is a very challenging problem. Certainly having just one metric is not acceptable for the purposes of our task, given all of the different properties or key regularities we would like our models to capture. The choice of right error metrics is critical, even more so in this domain, where problematic time series characteristics such as non-normalities or non-stationarities are often present in the data (Hewamalage et al., 2022). These characteristics can make some error measurements susceptible to break down, which can result in spurious conclusions about model performance. Determining which metrics are the most relevant or useful heavily depends on the problem(s) to be solved and a thorough understanding of the metrics' limitations. For example, to evaluate time series of social media activity, one might think that applying traditional and generally accepted regression metrics such as Root Mean Squared Error (RMSE) or Mean Absolute Error (MAE) would be appropriate. However, when the stated goal of a simulation or modeling effort is to help identify significant social phenomena such as anomalous activity periods (i.e., bursty behavior that might result from endogenous or exogenous influences), these metrics are clearly inadequate as time series that ignore the prediction of extreme values (outliers) would be favored. Figure 7 illustrates this observation. We show that a baseline auto-regressive model outperforms a machine learning XGBoost (Chen and Gestrin, 2016) model in both RMSE and MAE metrics despite predicting a relatively flat time series. In these cases, we must carefully consider metrics that account for the particular characteristics we want to model [e.g., volatility, skewness, Kleinberg's burst detection (Kleinberg, 2002), dynamic time warping (Berndt and Clifford, 1994)] instead of settling for metrics that, despite being widely used, do not align with the end goals.

Second, model accuracy is often evaluated in comparison with a baseline model's predictions: do its predictions beat the baseline's predictions. But what baseline is most useful for forecasting social media activity? Time series forecasting has been addressed in many contexts, from markets (Jiang, 2021) to spread of disease (Scarpino and Petri, 2019), and thus each context has its well-established baseline(s). But, social media activity reacts capriciously to both endogenous and exogenous events (Dutta et al., 2020; Horawalavithana et al., 2021), and thus baselines that are representative of processes in stock markets, for example, may be less representative of processes in social media domains. These observations motivated us to understand and document the relative performance benefits of existing time series forecasting methodologies for social media activity. For this, we defined a typical forecasting problem: forecasting the number of social media activities per hour within a discussion context for the next 168 h without using the ground truth information during the forecasting interval. We chose three existing techniques for forecasting time series: Shifted, that simply replays the past, Hawkes processes, and ARIMA. Our observations are presented in detail in a technical report (Ng et al., 2023), where we focused on answering various questions regarding the advantages and limitations of these baselines as applied to time series forecasting in social media.

**Figure 8:**
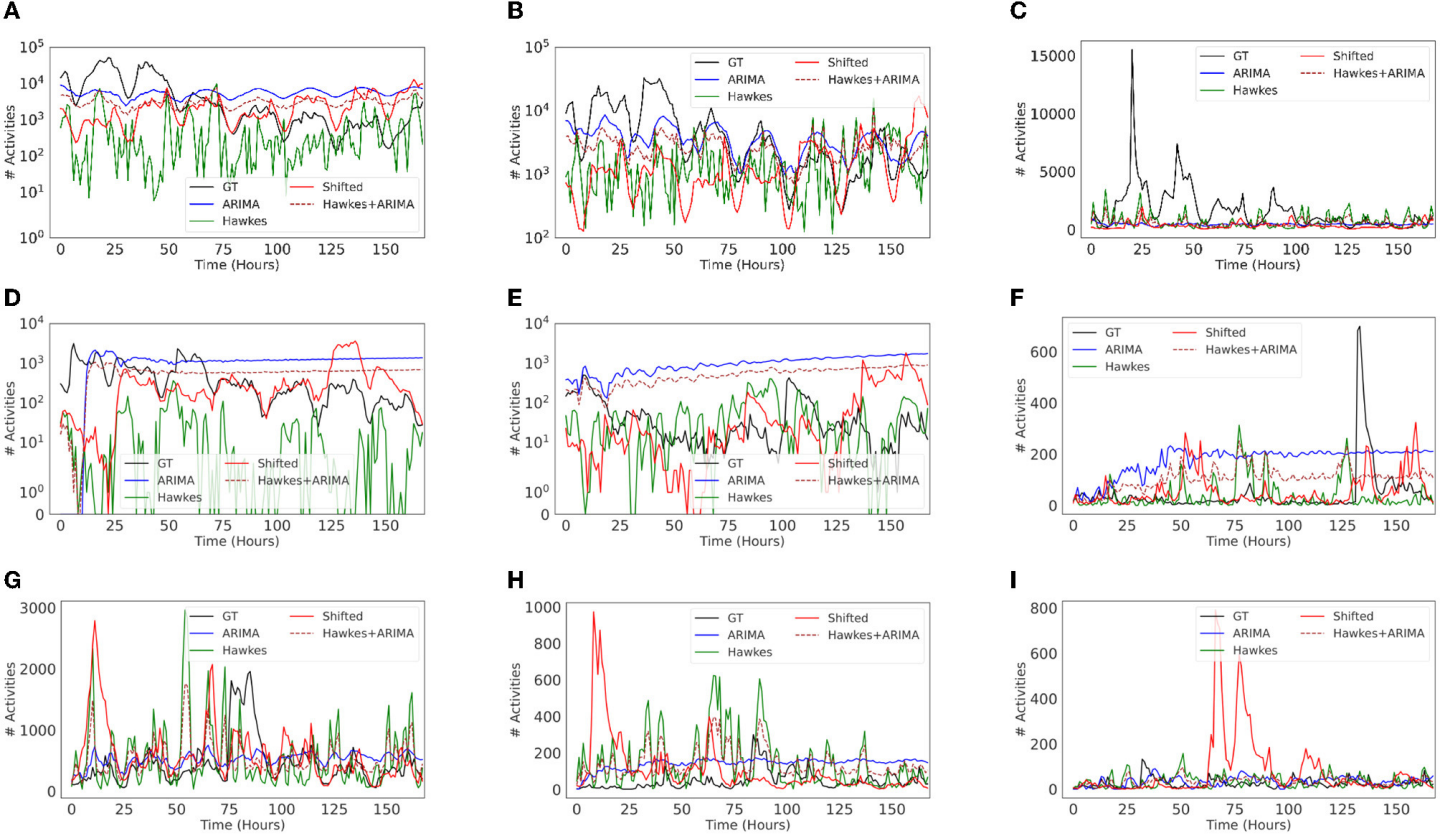
Predicted time series visualizations of different baseline regression models for Twitter activity. The top three most popular topics in each dataset are shown: Vz19 **(A–C)**, CPEC **(D–F)**, and BRIA **(G–I)**. Each baseline model was set to predict 1 week of activity at hour granularity (i.e., 168 time steps). **(A)** International/aid, **(B)** military, **(C)** maduro/dictator, **(D)** controversies/china/border, **(E)** leadership/sharif, **(F)** controversies/pakistan/baloch, **(G)** COVID, **(H)** travel, **(I)** mistreatment.

We found that ARIMA models tend to be inadequate or misleading in our context. When forecasting social media time series for long-term settings (e.g., days or weeks), ARIMA's predictions often fail to capture relevant temporal patterns observed in the ground truth. Figure 8 shows that ARIMA typically creates highly regular (and unrealistic) patterns of variation. On the other hand, the Shifted model, which simply replays the recent past, demonstrates competitive performance. Replaying the recent past is a cheap and highly reliable way to predict the near future in many scenarios and performance metrics. Another observation is that the choice of baselines also depends on the task at hand and what is the most important. For example, if modeling the final volume of information/message cascades, or predicting next day activity (assuming previous day ground truth data is always available), then ARIMA could still be a good choice. However, if the focus is to capture spiky behavior to identify, say, anomalous periods in the future, then comparing against baselines such as Shifted or Hawkes, or AR models that focus on variance/volatility (Lamoureux and Lastrapes, 1990) might be more appropriate/meaningful. Lastly, an ensemble of baselines could also result in more competitive models for comparison purposes. For example, when combining Hawkes and ARIMA by averaging, we observed an improvement for some topics in both volume of activity and temporal patterns.

## 6. Lessons on data use and relevance

As part of this DARPA-funded research project, we had access to a rich collection of well-curated longitudinal datasets from various platforms and contexts. For example, in the early stages of our research we had access to more than 10 years of activities on GitHub public repositories, 2 years of Reddit and Twitter conversations that mentioned software vulnerabilities, and over 1 year of discussion on the White Helmets. This variety of datasets from different platforms (Reddit, Twitter, and YouTube as often mentioned here, but also Jamii, GitHub, and Telegram) and highly variable socio-geo-political contexts, ranging from software development to international political contexts, allow us to make well-supported data-driven observations.

### 6.1. More (longitudinal) data is not always helpful

While the general assumption is that for machine learning (and especially deep learning) technologies the more data the better, we observed that this is not always the case with longitudinal data. Specifically, for simulation of temporal activity in social media more data from the distant past is likely not useful, due to various processes, such as platform algorithmic changes, topic evolution, or user population variations.

One example is the assignment of users to predicted events, as sketched in Section 4. In one solution, we assigned user identities to actions via a probabilistic approach that made use of a lookback parameter. This parameter was tunable and represented how much previous history must be considered to make predictions. Table 2 shows a series of experiments where we tested a range of lookback factor values to understand their impact measured on the user interaction network. Particularly, we looked at how much our models improved from the Shifted baseline in terms of the Relative Hausdorff Distance (RHD) between the predicted user interaction network (in this case, who is predicted to comment to whose message) and the ground truth network (who really comments on whose message). Again, the Shifted baseline replays the past 2 weeks of activities.

**Table 2:** YouTube user-assignment lookback factors vs. RHD distance percentage scores relative to how much improvement from shifted baselines.

**Lookback factor (in hours)**	**RHD PIFB score (%)**
96	19.26
120	19.49
144	19.54
168	19.21
192	19.52
216	18.98
240	18.91
480	18.46
720	18.27
960	17.48

We found that after a certain point (144 h in this example), using a longer history (manifested by a larger lookback parameter) did not lead to an increase in performance. The largest value we tested was 960 h (40 days) and it led to the worst result when compared to the other values. Moreover, as we increased the lookback factor, our user-assignment predictions became less accurate, thus affecting the overall performance in RHD. This highlights that using data from long ago compared to recent activity can negatively impact models' performance. Social media activity and user interactions are influenced by a combination of different processes such as changes in topics of discussion, or unseen factors such as exogenous activity or algorithmic biases. Thus, older patterns of activity (such as topic popularity) do not accurately reflect current trends and dynamics in social media behavior.

### 6.2. The power of exogenous vs. endogenous data

Ng et al. (2022) showed that models trained on endogenous activity can predict the final volume of activity more accurately than baselines. Table 3 shows the APE results across two datasets (Vz19 and CPEC) and two platforms (Twitter and YouTube) for one of our solutions (described in Ng et al., 2022) with three different variants: when only endogenous data is used for training the model (shown as Endogenous in the table), when both exogenous and endogenous data is used (Exogeneous) and an ensemble of models that takes the average over the predictions produced by the exogenous models (Ensemble) compared to three baselines. The model trained only on endogenous data was often ranked as one of the best models in terms of APE for volume of activities prediction, and in some cases it even outperformed the model trained on both endogeneous and exogeneous data. Despite their good performance on the total volume of activities as aggregated over time by APE, we found that the endogenous models were of limited benefit for capturing temporal patterns when real world events drove the social media activity.

**Table 3:** APE mean performance across topics of interest in two different challenges and two different platforms.

**Model**	**Vz19**		**CPEC**	
	**Twitter**	**YouTube**	**Twitter**	**YouTube**
Exogenous	**110.58**	92.96	**76.26**	**51.38**
Endogenous	**107.26**	**63.25**	**102.38**	53.16
Ensemble	**89.88**	**58.76**	**146.27**	**50.13**
ARIMA	302.76	79.61	236.93	87.12
Hawkes	124.76	80.36	187.58	**51.38**
Shifted	399.04	**51.73**	825.25	66.55

Top-3 best models are bold.

**Figure 9:**
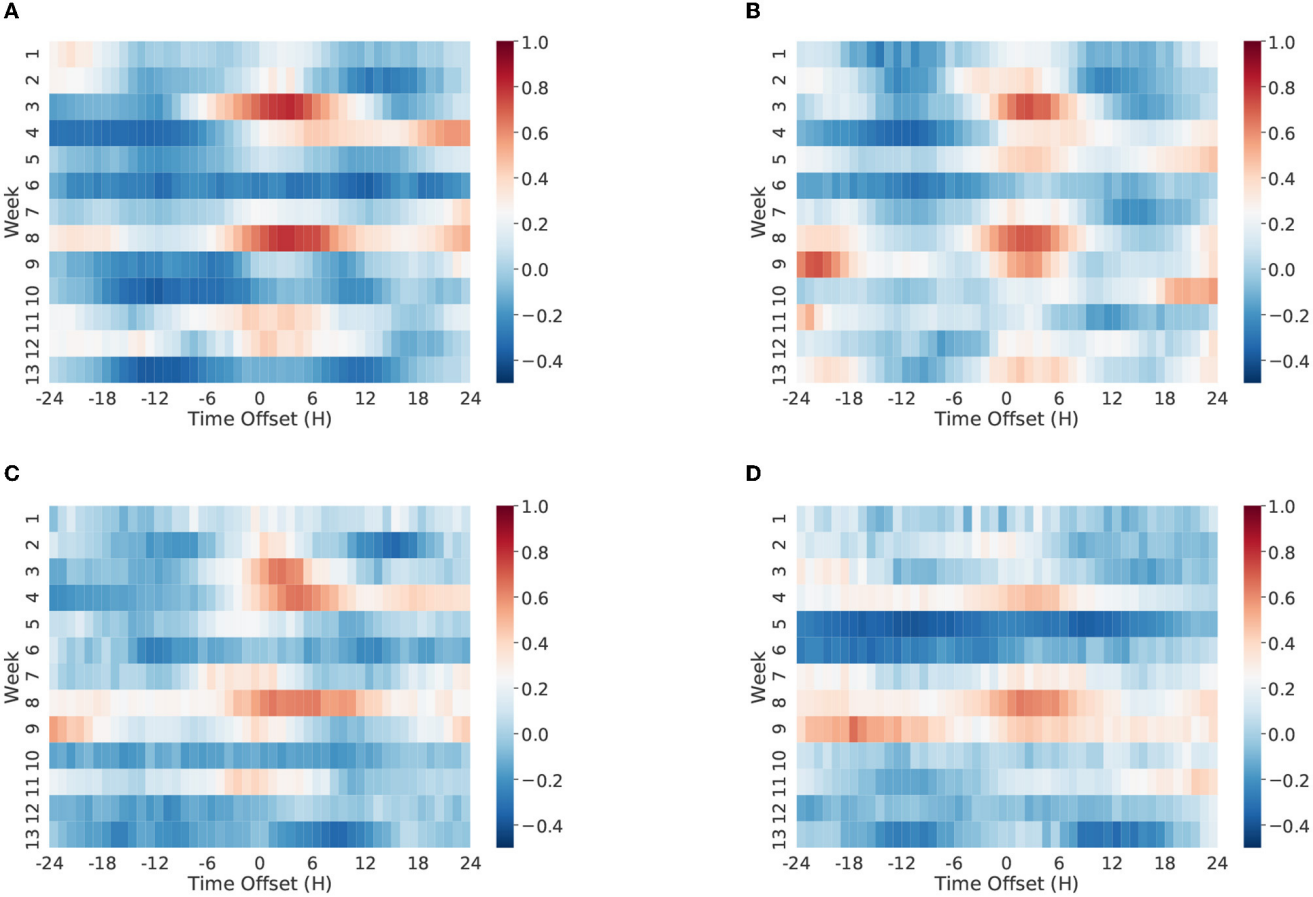
Time lagged correlation between GDELT and Twitter hourly activity timeseries. We split the hourly timeseries into equal chunks, where each chunk represents the hourly timeseries in a week. We calculate the Pearson correlation coefficient between GDELT and Twitter activity timeseries in each week. We incrementally shift one time series vector by hours and repeatedly calculate the correlation between two timeseries. The correlation values represented in the positive time offsets suggest that GDELT reacts to Twitter, and the correlation values represented in the negative time offsets suggest that Twitter reacts to GDELT. The cell colors in the heat map represent the correlation coefficient values as presented in the color bar. The numbered weeks in the y-axis are relative to the start date of January 01, 2019. **(A)** Anti-maduro (Spanish), **(B)** pro-maduro (Spanish), **(C)** anti-maduro (English), **(D)** pro-maduro (English).

For example, Figure 9 shows a time-lagged correlation analysis on the Vz19 dataset. Our results suggest that, at particular periods of time, the volume of Twitter discussions about the Venezuelan political conflict were highly correlated with previous real world events as captured in GDELT. We also observed that distinct groups or communities tended to react differently to exogenous influences.

These observations suggest that taking into account exogenous influences is paramount for accurately forecasting the activity of different subjects of discussion, especially for capturing sudden peaks of activity. In fact, we showed the importance of incorporating exogenous features in Ng et al. (2021a). Via experimental evaluations on two Twitter datasets, we demonstrated that models that did not consider exogenous features often predicted flat lines of activity or time series patterns that tended to regress toward the mean. Such predictions are not useful in operational scenarios where forecasting anomalous periods of activity over time is valuable.

### 6.3. Augmenting training data can be useful for increasing model accuracy

While longer historical data is not always beneficial in terms of forecasting the future, we often found ourselves wanting more contemporary data than there was available, such as more activities in a particular interval on a particular topic and platform. To satisfy similar needs, data practitioners often attempt to augment datasets via synthetically generated data. We tested this approach in our problem setup.

**Figure 10:**
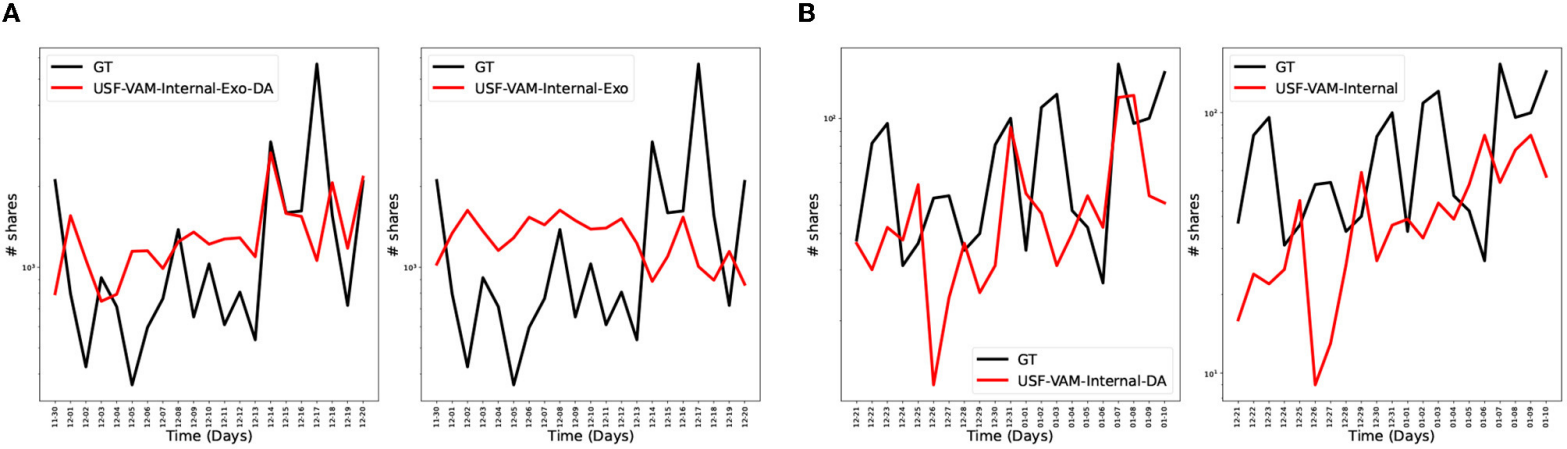
Some instances in which the data-augmented models outperformed the non-data-augmented models. The red curves represent the predictions and the black curves represent the ground truth. In each sub plot, the left sub plot represents the data-augmented predicted time series, while the right sub plot represents the non-data-augmented predicted time series. As one can see in each subfigure, the data-augmented models matched the ground truth better than their non-data-augmented counterparts. **(A)** Anti—VAM data augmented (Left) vs. VAM non-data-augmented (Right). **(B)** other-state-affiliated-accounts—VAM data augmented (Left) vs. VAM non-data-augmented (Right).

We augmented data by using a noise based approach applied to all data from a topic. Gaussian noise was applied to the original feature vectors to obtain the augmented ones. Figure 10 shows an example of how data augmentation can provide benefits. In Figure 10A, a prediction from a data-augmented model is compared to a prediction from the same model trained on the original data for the topic *anti* of the BRIA dataset. The NRMSE and APE for the data-augmented model are 0.12 and 0.23, respectively, while the NRMSE and APE for the non-data-augmented model are 0.16 and 3.8, respectively.

In Figure 10B, the data-augmented and non-data-augmented model predictions were compared on a different the *other-state-affiliated-accounts* topic, again on Twitter BRIA dataset. The NRMSE and APE for the augmented model are 0.03 and 31.71, respectively. For the non-data-augmented model, the NRMSE and APE are 0.06 and 43.53, respectively. Overall, we found that data augmentation is useful for topics with very sparse time series. Particularly, augmentation made these models more robust to small fluctuations and improved the generalization to unseen data, leading to more accurate predictions.

## 7. Observations on forecasting social media activity

While previous sections discussed lessons learned from working with large and varied datasets as applied to designing and evaluating machine learning-based models capable of forecasting social media activity from micro to macro level, this section discusses the challenges inherent to the problem rather than stemming from our particular solutions. In hindsight, these challenges seem intuitive. Stating them explicitly might help future research focus on the most difficult problems first.

### 7.1. Long-term forecasting requires contemporary exogenous data

Long-term forecasting refers to a stream of discrete counts that must be predicted without any knowledge/feedback of the ground truth or updated exogenous data and not to the duration of the predictions (e.g., weeks, months, years). Our experience taught us that endogenous signals alone (historical platform activity) do not produce reliable forecasts very far into the future. Not only can they not reliably capture activity burst behavior due to exogenous events, but when predicting the future relies on a recent past that is also in the predicted future, solutions that feed predictions back into training data lead to accumulation of errors.

**Figure 11:**
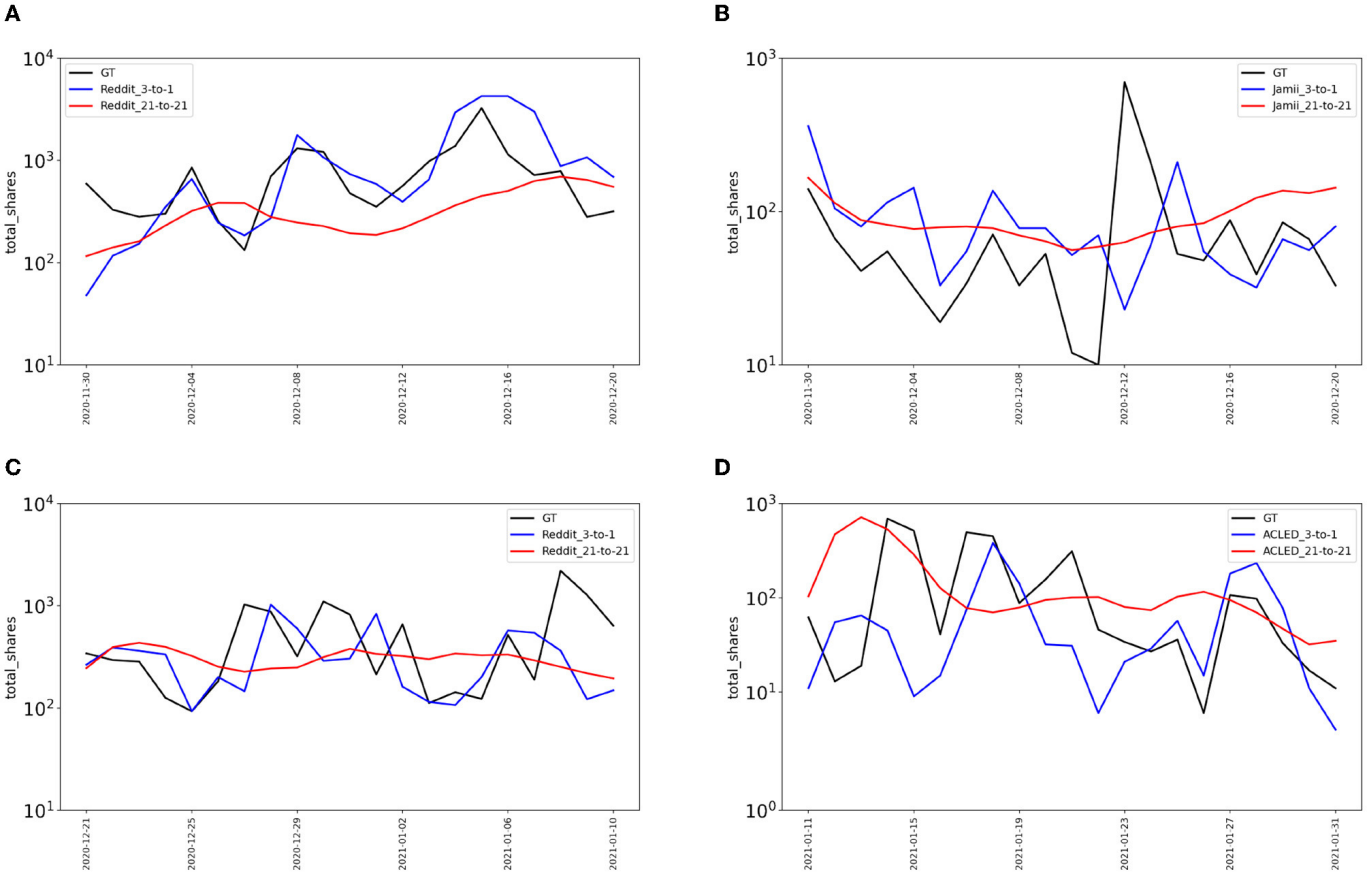
Sample time series visualizations on Twitter activity from the BRIA dataset. The blue lines are our LSTM-based models presented in Ng et al. (2022) and trained with recent exogenous sources (3 previous days), the red lines are our models trained with distant data from exogenous sources (21 previous days), and the black lines are the ground truth. **(A)** COVID, **(B)** environmentalism, **(C)** mistreatment, **(D)** UN.

The introduction of exogenous features into our models alleviates these issues. But, it is important to emphasize that as we stretch out the number of future time steps for which prediction is required, flat predictions can result even if exogenous data is available. Figure 11 shows various visualizations of predicted time series on the BRIA dataset that illustrate this lesson. We observe that when considering recent exogenous signals (e.g., from the previous 3 days) and shorter future steps (next day prediction) at day granularity (labeled “3-to-1” in Figure 11), our models can generally capture spikes of activity and temporal trends more accurately. On the other hand, for longer multi-step ahead predictions and old exogenous information history (“21-to-21”, corresponding to 21 days of history and 21 days prediction in the future), the time series are almost flat and in many cases regress toward the mean.

### 7.2. Macro-level characteristics are easier to predict than micro-level

This observation was independently reached by Bollenbacher et al. (2021) and simply states that predicting the number of posts or the number of users who post on a topic is easier to get right than predicting who are the users who post, or even what are the general characteristics of their interaction patterns. We illustrate this lesson by reporting the percent improvement from the Shifted baseline when performance is measured in APE of volume of activities or network metrics. For two families of models presented in Mubang and Hall (2022) and Ng et al. (2022), we observed that the relative improvement over the Shifted baseline when measuring the volume of activities on the BRIA dataset is ranging between 35.6 and 45.99%. However, the percentage improvement over the network characteristics of the user interaction network against the same Shifted baseline are ranging between 14.55 and 29.61%.

Another example where groups are more predictable than individuals is at the level of diffusion cascades. Horawalavithana et al. (2022) proposed a probabilistic generative model with the support of a genetic algorithm and LSTM neural networks to predict the growth of discussion cascades on Reddit. Our approach focused on predicting the microscopic properties of a *pool* of conversations, and thus it modeled groups of conversations instead of individual conversations.

We demonstrated that our solution predicts the properties of the resulting cascades more accurately than the baselines. However, while our solution more accurately traces both the distribution of conversation sizes and that of conversation viralities than any of the baselines, we found that it struggles with the end points of the spectrum: the properties of the very small and very large conversations. These observations highlight the difficulty of predicting the effect of a particular message as opposed to a group of messages. The finer granularity predictions require finer granularity features that we cannot possibly observe and thus use, such as a particular user's attention to a topic or even to the social media platform at any time.

## 8. Summary and open problems

This paper presents an overview of our data-driven observations from developing social media forecasting techniques over a 4-year project. While many of the models developed and tested were published elsewhere (Liu et al., 2019; Horawalavithana et al., 2022; Mubang and Hall, 2022; Ng et al., 2022), this paper synthesizes our conclusions from working with more than 5 years of aggregated social media data from three different contexts.

Our main observations include the following. First, long-term forecasting of social media activity at fine granularity is a scarcely researched topic despite its significant potential for generating realistic synthetic longitudinal traces for research; enabling safe experimental environments for testing intervention techniques; and identifying potentially inorganic behavior that significantly diverges from predicted trends. Most research to date focused on predicting the characteristics of a future point in time rather than a sequence of timed events with authorship attributions (“who does what when”).

Second, research challenges in this space are more significant the more finer the granularity of the desired prediction is. Thus, predicting which user reacts to which piece information at what time is perhaps close to impossible given the limited information available from social media platform activities, yet predicting the number of users engaged with a topic over time is more doable. Many applications can be beneficial even with such limitations. Overcoming these limitations, however, may pose significant user privacy concerns and safety risks, which is not what we advocate.

Third, exogenous information is paramount for predicting the evolution of social media activity, given how interconnected the digital and the non-digital worlds are. Which exogenous sources of information are relevant for which topic of discussion, however, are not constant over time and different contexts. For example, we found Reddit data to be useful for forecasting Twitter activity for several topics related to Venezuela political crisis of 2019 while ACLED and GDELT data for forecasting Twitter activity for topics related to CPEC.

Fourth, which performance metrics best capture the success in forecasting tasks is dependent on the objective to which forecasting is to be used. For example, if the objective is to identify the overall volume of posts related to a topic over a period of time vs. predicting spikes of activity over a short period of time, different performance metrics should be chosen, as well as different baseline techniques.

Finally, our experience showed that end-to-end machine-learning solutions are less accurate than decomposing the problem into specialized models able to correct unrealistic combined predictions, such as more users who post than the number of messages posted in a given interval.

In addition to improving solutions in this problem space, future research directions include developing simulation scenarios based on forecasting social media activity. Currently, social media platforms are solely responsible for containing the damaging activities on their platforms, especially related to the diffusion of information, yet they are totally opaque in terms of the measures they implement or the scientific support for such measures. Our simulators could be used to evaluate the impact of interventions targeted at addressing issues such as polarization, diffusion of information, or the deterrence of coordinated content promotion efforts. Intervention techniques for testing may include censoring user accounts, censoring content (URLs, particular tweets, domains, hashtags), censoring narratives, and scrambling stances such that a user appears to post opposing viewpoints toward particular topics.

## Data availability statement

The data that support the findings of this study are available from the authors but restrictions apply to the availability of these data, which were used under license for the current study, and were collected by Leidos.

## Author contributions

All authors listed have made a substantial, direct, and intellectual contribution to the work and approved it for publication.

## Footnotes

^1^ https://twitter.com

^2^ https://instagram.com

^3^ https://tiktok.com

^4^ https://youtube.com

^5^ https://github.com

^6^ https://www.forbes.com/sites/petersuciu/2021/11/24/youtube-removed-dislikes-button–it-could-impact-how-to-and-crafts-videos/

^7^ https://www.businessinsider.com/why-facebook-didnt-make-dislike-button-2016-2

^8^ https://www.pewresearch.org/internet/fact-sheet/social-media/

^9^ https://www.nytimes.com/2018/08/06/technology/china-generation-blocked-internet.html

^10^ https://afritechnews.com/jamiiforums/

^11^ https://www.darpa.mil/program/computational-simulation-of-online-social-behavior

^12^ https://www.nytimes.com/2019/04/30/world/americas/venezuela-crisis.html

^13^ https://www.cfr.org/blog/china-pakistan-economic-corridor-hard-reality-greets-bris-signature-initiative

^14^Jamii forums is a social networking site popular in East and Central African countries.

^15^ https://github.com/socsim-ta2/socialsim_package/blob/master/socialsim/twitter_cascade_reconstruction.py

^16^ https://github.com/pnnl/socialsim_package
